# 5-Hydroxymethylcytosine: Far Beyond the Intermediate of DNA Demethylation

**DOI:** 10.3390/ijms252111780

**Published:** 2024-11-02

**Authors:** Kaixi Zheng, Zhengbing Lyu, Jianqing Chen, Guodong Chen

**Affiliations:** 1 College of Life Sciences and Medicine, Zhejiang Provincial Key Laboratory of Silkworm Bioreactor and Biomedicine, Zhejiang Sci-Tech University, Hangzhou 310018, China; 2023210901110@mails.zstu.edu.cn (K.Z.); zhengbingl@zstu.edu.cn (Z.L.); cjqgqj@zstu.edu.cn (J.C.); 2 School of Life Sciences, Central South University, Changsha 410031, China

**Keywords:** 5hmC, 5mC, demethylation, TET, transcription regulation

## Abstract

Epigenetics plays a pivotal role in regulating gene expression and cellular differentiation. DNA methylation, involving the addition of methyl groups to specific cytosine bases, is a well-known epigenetic modification. The recent discovery of 5-hydroxymethylcytosine (5hmC) has provided new insights into cytosine modifications. 5hmC, derived from the oxidation of 5-methylcytosine (5mC), serves as both an intermediate in demethylation and a stable chemical modification in the genome. In this comprehensive review, we summarize the recent research advancements regarding the functions of 5hmC in development and disease. We discuss its implications in gene expression regulation, cellular differentiation, and its potential role as a diagnostic and prognostic marker in various diseases. Additionally, we highlight the challenges associated with accurately detecting and quantifying 5hmC and present the latest methodologies employed for its detection. Understanding the functional role of 5hmC in epigenetic regulation and further advancing our understanding of gene expression dynamics and cellular processes hold immense promise for the development of novel therapeutic strategies and precision medicine approaches.

## 1. Introduction

Epigenetics encompasses the study of heritable phenotype changes that occur without alterations to the DNA sequence. It exerts a pivotal influence on gene expression regulation, cellular differentiation, fate determination, environmental responses, and adaptability within living organisms. Among the diverse array of epigenetic modifications, DNA methylation stands out as a prominent mechanism. This process involves the transfer of methyl groups from S-adenosylmethionine (SAM) by methyltransferases onto specific cytosine bases. Within the mammalian genome, cytosine (C) at the 5th position of the pyrimidine ring in CpG dinucleotides is particularly prone to undergoing methylation. 5-methylcytosine (5mC), recognized as the fifth DNA base, assumes a critical role in the regulation of gene expression, cellular differentiation, genome stability, and responsiveness to environmental cues. Numerous studies have demonstrated that 5-methylcytosine (5mC) can undergo oxidation catalyzed by ten-eleven translocation (TET) enzymes, resulting in the formation of 5-hydroxymethylcytosine (5hmC). Subsequently, 5hmC can undergo further oxidation to generate 5-formylcytosine (5fC) and 5-carboxylcytosine (5caC) [1,2,3]. Notably, 5hmC serves not only as an intermediate product in the demethylation process of 5mC but also exhibits distinct distribution patterns and stable levels. It represents a durable chemical modification in the genome, playing a crucial role in the regulation of gene expression and the field of epigenetics. Consequently, 5hmC is often referred to as the sixth DNA base.

## 2. The Discovery of 5hmC: From Bacteriophages to Epigenetic Landscapes

5mC has been extensively investigated as a prominent epigenetic modification, playing a well-established role in gene expression regulation, cellular differentiation, and disease development. However, through further exploration of 5mC, researchers achieved a remarkable breakthrough by uncovering a novel cytosine modification variant termed 5hmC within bacteriophage genomes [4]. This modification entails the addition of a hydroxyl group to the methyl group bound to the 5th carbon of the cytosine ring through the oxidation of the methyl group, expanding our understanding of the diverse repertoire of cytosine modifications.

5hmC in DNA was initially observed in specific bacteriophages. In 1952, Wyatt and Cohen made the seminal discovery of 5hmC in *E. coli* bacteriophages [4]. Subsequently, in 1953, the existence of 5hmC in bacterial and animal viral nucleic acids was further confirmed [5]. Within the T4 bacteriophage, an essential component of the bacteriophage DNA protection system, beta-glucosyltransferase (BGT), facilitates the transfer of glucose (Glc) from uridine diphosphate glucose (UDP-Glc) to 5hmC, thereby providing resistance against host nucleases [6]. In 1972, N. Penn and colleagues detected approximately 15% of total cytosine bases as 5hmC in DNA isolated from adult rats. Moreover, the presence of 5hmC was also identified in DNA hydrolysates from mouse and frog brains, as well as rat brain and liver RNA [7].

However, significant progress in research on 5hmC remained limited until a groundbreaking discovery in 2009 by Tahiliani and colleagues, who demonstrated the widespread presence of 5hmC in mouse embryonic stem cells [1]. In the same year, S. Kriaučionis and Heinz employed thin-layer chromatography to identify a substantial amount of 5hmC in Purkinje neurons, characterized by large nuclei and low levels of euchromatin [8]. Subsequent investigations further affirmed the presence of 5hmC in all tissues and cell types in mice, with particularly elevated levels observed in the central nervous system (CNS) [9]. In 2011, the tissue-specific distribution of 5hmC in humans was established, revealing heightened levels in the brain and liver, moderate levels in kidney or heart cells, and relatively lower levels in spleen or thymus tissues, accounting for only 5–15% of CNS tissue levels [10].

Another significant milestone in advancing our understanding of 5hmC was the discovery of the TET protein family. Tahiliani et al. made the pivotal observation that the TET1 protein, known as the fusion partner of the MLL gene in acute myeloid leukemia, exhibited the ability to catalyze the conversion of 5mC to 5hmC in vitro. Notably, TET1 functions as an enzyme reliant on 2-oxoglutarate (2OG) and Fe(II) [1]. A subsequent report in 2011 highlighted that TET proteins could further convert 5mC to 5fC and 5caC, emphasizing the potential impact of dysregulated TET proteins on the levels of 5hmC, 5fC, and 5caC within the genome [2,3]. Furthermore, subsequent research endeavors identified thymine-DNA glycosylase (TDG), a specific enzyme capable of recognizing and removing 5caC, thereby providing valuable insights into the active demethylation pathway [2].

**Figure 1:**
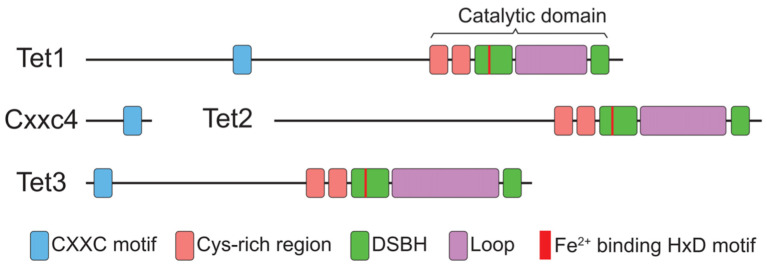
The structures of TET1, TET2, and TET3. All TET proteins possess a catalytic core consisting of a cysteine-rich region and a double-stranded β-helix domain, enabling the binding of Fe^2^⁺ and α-ketoglutarate. TET1 and TET3 contain a CXXC domain for recognizing unmethylated CpG sites, whereas TET2 lacks this domain and relies on interactions with other proteins.

Research has found that TET1, TET2, and TET3 are key proteins involved in DNA demethylation. Although they share some structural and functional similarities, there are significant differences, particularly in their domains, functions, and regulatory mechanisms. Structurally, the catalytic core of the TET family consists of two primary regions: a cysteine-rich region and a double-stranded β-helix domain (DSBH). Together, these regions form the catalytic center of the TET proteins, which bind Fe^2^⁺ and α-ketoglutarate (α-KG) to catalyze the oxidation of 5mC. This core structure is conserved across all three TET proteins. However, there are important structural differences. For example, TET1 and TET3 contain a CXXC domain, which recognizes unmethylated CpG sites and guides DNA binding. TET2, on the other hand, lacks a CXXC domain and instead relies on interactions with other proteins to localize to its target DNA [11] (Figure 1).

Functionally, TET1 is primarily expressed in embryonic stem cells and undifferentiated cells, where it is associated with gene promoter regions. TET1 plays a key role in regulating gene expression by maintaining 5hmC levels, which is particularly important for preserving pluripotency and guiding developmental processes [12]. In contrast, TET2 is crucial in the hematopoietic system, especially in regulating the differentiation of hematopoietic stem cells. Mutations in TET2 are strongly linked to various hematological cancers, such as acute myeloid leukemia (AML) and myelodysplastic syndromes (MDS) [13]. TET3, on the other hand, plays a significant role during development, being highly expressed in early embryogenesis and the nervous system. During early embryonic development, TET3 is involved in the demethylation of the paternal genome and regulates gene expression during neuronal differentiation [14].

## 3. 5hmC and Active DNA Demethylation

5mC exhibits relative chemical and genetic stability. The process of de novo DNA methylation is primarily carried out by two DNA methyltransferases: DNMT3A and DNMT3B. During this process, DNMT3A and DNMT3B add methyl groups to the cytosine residues of newly synthesized DNA, establishing new methylation patterns that are essential for gene expression control and cellular differentiation [15]. Once the de novo methylation is established, the maintenance of these methylation marks during DNA replication is handled by DNMT1. As cells divide, DNMT1 ensures that the newly synthesized daughter strands inherit the methylation patterns from the parent strands. It recognizes hemi-methylated DNA, wherein one strand is methylated while the complementary strand remains unmethylated, and subsequently adds methyl groups to the unmethylated cytosines on the daughter strand. This maintenance mechanism is vital for the stable transmission of epigenetic information across generations of cells, thereby preserving gene regulation and genome integrity throughout cellular replication [16].

The active demethylation pathway of 5mC was initially identified in plants. In Arabidopsis, the Demeter family of DNA deoxyribonucleases efficiently eliminates 5mC, leading to the formation of a site with a missing base on the DNA chain. This process results in DNA demethylation and the activation of transcription in target genes [17]. However, in mammals, the pathway of 5mC demethylation can be categorized into two distinct types: replication-dependent 5mC dilution, also known as passive demethylation, which inhibits the maintenance of DNA methylation mechanisms (UHRF1/DNMT1) [18], and active DNA demethylation in mammals which is mediated by TET enzymes [1,2,3].

Replication-dependent 5mC dilution, commonly referred to as passive demethylation, describes the gradual loss of DNA methylation marks during replication. In this process, newly synthesized daughter strands are not methylated unless the maintenance methylation machinery, particularly DNMT1, is active. If DNMT1’s function is compromised or absent, this maintenance mechanism fails, leading to an accelerated loss of DNA methylation marks on the daughter strands. This process occurs spontaneously during replication and does not require additional enzymatic involvement, making it a critical aspect of epigenetic regulation in response to changes in the cellular environment [19]. However, the primary focus of this paper is on TET-mediated active DNA demethylation.

The first evidence supporting active DNA demethylation in mammals was presented in a study published by W. Mayer in 2000. Immunostaining revealed that demethylation occurs in the maternal genome of mouse embryos after several rounds of cell division, while a noticeable decrease in DNA methylation signal occurs in the paternal genome 6–8 h after fertilization, prior to DNA replication initiation. Five years later, H. Morgan confirmed the rapid erasure of paternal imprints through DNA demethylation in the embryos of mid-gestation mammals [20].

With advancements in research, the complex process of active demethylation of 5mC has become increasingly well understood. In mammals, TET1, TET2, and TET3 serve as the key enzymes responsible for this demethylation process. They initiate the conversion of 5mC to 5hmC, which can then be further oxidized to form 5fC and 5caC [2,3]. Once 5fC or 5caC is generated, DNA repair mechanisms step in to remove these oxidative products. TDG, a critical enzyme in this process, recognizes and excises 5fC and 5caC, leaving behind an abasic site (AP site) [2,21]. The resulting AP site is then repaired through the base excision repair (BER) pathway, where newly synthesized nucleotides replace the nucleotides at these sites, restoring them to unmodified cytosine [22]. In 2016, Weberzai demonstrated through in vitro biochemical reconstruction that the TET–TDG–BER pathway effectively converts 5mC to unmodified cytosine, highlighting the tight coupling among these three components to minimize the possibility of double-stranded breaks during the demethylation process [22].

In recent years, as researchers have gained deeper insights into methylation and demethylation pathways, additional potential mechanisms have been proposed. For instance, some scientists speculate that 5caC could be directly converted into C through a decarboxylase enzyme, bypassing the BER pathway. However, no clear evidence has been found to confirm the presence of such a decarboxylase in mammalian cells [23]. Also, some researchers have suggested the existence of a group of enzymes involved in the deamination of cytosine, known as AID and APOBEC. These enzymes can catalyze the conversion of the amino group of 5hmC to a carbonyl group, resulting in the formation of 5-hydroxymethyluracil (5hmU) [24]. The generated 5hmU can then undergo conversion to unmodified cytosine through the previously mentioned TDG–BER pathway.

**Figure 2:**
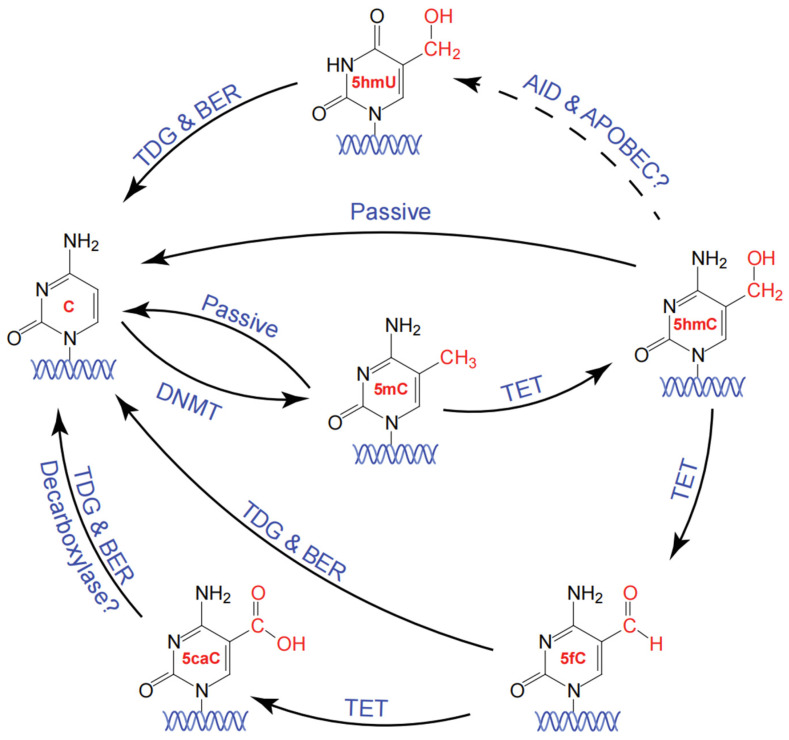
The cycle of DNA methylation and demethylation. DNA methyltransferases (DNMTs) convert unmodified cytosine into 5-methylcytosine (5mC). Subsequently, through the action of ten-eleven translocation (TET) enzymes, 5mC is oxidized to 5-hydroxymethylcytosine (5hmC). TET enzymes further oxidize 5hmC to 5-formylcytosine (5fC) and 5-carboxylcytosine (5caC). Both 5fC and 5caC can be restored to unmodified cytosine through the TDG–BER pathway.

In conclusion, the pathways of methylation and demethylation are complex and diverse, encompassing various enzymes and mechanisms (Figure 2). These mechanisms involve not only the classical DNMT methyltransferases and demethylating TET enzymes, but also other enzymes involved in repair and metabolism, such as TDG and the AID/APOBEC family. As research techniques advance in the future, it is likely that more novel mechanisms will be discovered, further elucidating the significant roles that methylation and demethylation play in biological processes [25].

## 4. Advances and Challenges in Detecting 5hmC at the Genomic Level

The detection of 5hmC at the genomic level poses a significant challenge due to its low abundance [26]. Complicating matters further, 5hmC bears a close chemical resemblance to 5mC, differing only by the addition of a hydroxyl group (-OH) to the methyl group of 5mC, making their discrimination challenging.

Traditional detection methods, such as thin-layer chromatography (TLC) [27] and mass spectrometry (MS) [28], have been employed for global quantification of nucleotide levels. These techniques are advantageous due to their relative simplicity and ability to provide overall 5hmC content data. However, these methods have notable limitations, as they cannot provide genomic localization information for 5hmC and lack specific sequence context.

With the continuous advancement and refinement of technologies, innovative detection methods have emerged. These methods encompass chemical modification-based enrichment and sequencing techniques, such as TET-assisted bisulfite sequencing (TAB-seq) [28] and methylation-sensitive restriction enzyme combined with sequencing (MRE-seq) [29]. In TAB-seq, 5hmC is protected from further oxidation by being modified to β-glucosyl-5-hydroxymethylcytosine (5gmC), preventing TET enzymes from oxidizing it to 5caC. This protection step ensures that during bisulfite conversion, 5mC is converted to 5fC and then uracil (U), while 5hmC remains unchanged, allowing its precise detection after sequencing [30].

Furthermore, several cutting-edge technologies have integrated high-throughput sequencing to investigate 5hmC and its derivatives at the genomic level. Noteworthy examples encompass hydroxymethyl-DNA immunoprecipitation sequencing (hMeDIP) [31], chemical labeling [32], and single-molecule real-time (SMRT) sequencing [33]. Among these, hMeDIP is a technique that combines DNA immunoprecipitation with high-throughput sequencing. It involves immunoprecipitation of fragmented genomic DNA, followed by purification and subsequent high-throughput sequencing analysis, enabling the generation of comprehensive distribution maps and relative abundance information of 5hmC in the genome [31]. However, due to its reliance on antibody specificity, this method has relatively low resolution and cannot achieve single-base precision. Additionally, hMeDIP only provides relative abundance information and cannot precisely quantify 5hmC levels. Therefore, while hMeDIP is suitable for large-scale screening, it is not ideal for the high-precision quantification of 5hmC.

Recent advancements in third-generation sequencing technologies, such as single-molecule real-time (SMRT) sequencing by Pacific Biosciences (PacBio) and nanopore sequencing by Oxford Nanopore Technologies (ONT), have overcome the read-length limitation and offer ultra-long read, single-base resolution at a genome-wide scale [34]. Nanopore sequencing, in particular, directly detects DNA translocation through a voltage-biased nanopore sensor, allowing for rapid sequencing with single-molecule sensitivity [35]. Compared to traditional TAB-seq and hMeDIP, nanopore sequencing does not rely on PCR amplification or chemical modifications, thereby preserving the native state of DNA. This technology theoretically allows for the simultaneous detection of both 5mC and 5hmC, providing modification distribution information across the entire genome. However, nanopore sequencing still faces some challenges, particularly in regions with low CG density or complex repetitive regions, where its detection accuracy needs further optimization.

When selecting a 5hmC detection technology, researchers need to weigh their options based on the specific requirements of their experiments. If high precision and single-base resolution are required, TAB-seq is undoubtedly an ideal choice, although it comes with high technical complexity and costs. For large-scale screening or exploratory studies, hMeDIP provides a more convenient and cost-effective option, but its lower resolution makes it difficult to accurately locate 5hmC. Chemical labeling techniques are easy to operate and suitable for rapid detection, but they may be slightly inferior in terms of specificity and sensitivity. Third-generation sequencing technologies, although still in the optimization stage, have shown great potential, especially in the direct detection of native DNA modifications.

Although current detection technologies have made advancements in sensitivity and specificity, the detection of 5hmC still faces multiple challenges. For instance, existing antibodies exhibit variations in sensitivity and specificity, which may lead to false-positive or false-negative results. During sample processing, the degradation of 5hmC or its conversion to 5mC can also impact the accuracy of the detection results. Furthermore, even when 5hmC is successfully detected, interpreting its biological significance remains a crucial issue, as its specific functions in different biological contexts are not yet fully understood. Therefore, overcoming these challenges requires technological innovation, standardized detection methods, and in-depth biological research to gain a more comprehensive understanding of the importance of 5hmC as an epigenetic mark.

## 5. Distribution, Function, and Dynamics of 5hmC

Through the application of existing technologies, several characteristics of 5hmC have been unveiled. By employing stable isotope labeling of cytosine derivatives in mammalian cell DNA and employing ultra-sensitive tandem liquid chromatography-mass spectrometry, it has been discovered that the majority of 5hmC is not a transient intermediate but rather a stable epigenetic modification. Furthermore, a correlation has been established between global levels of 5hmC and cellular proliferation [36]. Notably, 5hmC is found at replication origins in mammals and exerts a negative influence on cell division by prolonging the duration of the G1 phase [37]. These findings suggest that 5hmC not only maintains consistent levels throughout the cell cycle but also potentially serves a regulatory role in cell cycle processes.

CpG sites are dinucleotides consisting of a cytosine nucleotide followed by a guanine nucleotide, connected by a phosphate bond. These sites are frequently targeted for DNA methylation, which plays a crucial role in gene regulation. CpG islands, which are DNA regions relatively enriched in CpG sites compared to their surroundings, often reside in gene promoter regions and maintain an unmethylated or low-methylated state, thereby contributing to the preservation of normal gene expression. Consequently, CpG islands frequently exhibit enrichment of 5hmC [38], which is associated with the active transcriptional state of genes. Studies have revealed that numerous genomic regions or CpG sites undergo modification by 5hmC in mouse embryonic stem cells (ESCs) [39], and the binding affinity of TET1 positively correlates with CpG density [40]. TET1 binds to CpG-rich regions through its CXXC domain to impede interference from DNA methyltransferases. Through its hydroxylase activity, TET1 controls DNA methylation by converting 5mC to 5hmC [41]. Moreover, TET1 fine-tunes transcription and counteracts abnormal DNA methylation in CpG-rich sequences, thereby contributing to the regulation of DNA methylation fidelity [42]. In vitro analyses have demonstrated that human TET2 exhibits diminished activity towards 5mCpC and 5mCpA compared to 5mCpG, potentially due to impaired base-stacking interactions [43]. The distribution patterns of 5fC/5caC and 5hmC suggest a potential association between the catalytic process of TET enzymes and local chromatin accessibility [44].

The distribution of 5hmC is primarily observed in gene promoter regions and transcriptional regulatory regions. Chromatin immunoprecipitation combined with sequencing (ChIP-seq) analysis conducted by Williams in mouse embryonic stem cells (ESCs) has revealed the enrichment of TET1 at CpG islands, active promoters, and bivalent promoters. The binding affinity of TET1 exhibits a positive correlation with CpG density and H3K4me3 levels [42]. Additionally, 5hmC demonstrates enrichment at transcriptional enhancers [39], DNase hypersensitive sites, and regions surrounding transcription factor binding sites [28]. In both ESCs and neuronal cells, 5hmC is notably enriched in euchromatic regions while exhibiting lower levels in heterochromatic regions [45]. Notably, the levels of 5hmC display an increasing trend from transcription start sites (TSSs) to transcription termination sites (TTSs), suggesting a potential association between 5mC oxidation and transcriptional elongation [46].

The distribution of 5hmC demonstrates pronounced cell and tissue specificity. It exhibits relatively high levels in brain, liver, kidney, and colon tissues (0.40–0.65%), lower levels in lungs (0.18%), and very low levels in the heart, mammary glands, and placenta (0.05–0.06%) [10]. In Purkinje neurons, 5hmC constitutes approximately 40% of total cytosine modifications, showing an enrichment nearly 10 times higher than that in mouse embryonic stem (mES) cells, while its content is lower in other tissues [8]. Additionally, 5hmC not only exhibits tissue specificity but also displays dynamic characteristics. During the early development of embryonic stem cells, 5hmC levels are low, potentially associated with the preservation of cellular pluripotency and differentiation potential [47]. As cells differentiate into distinct cell types, 5hmC levels undergo dynamic changes. For instance, Szulwach conducted whole-genome mapping of 5hmC in the mouse hippocampus and cerebellum and observed a programmed acquisition of 5hmC in neuronal cells from neurodevelopment to adulthood [48]. Moreover, the dynamic changes in 5hmC may be linked to the occurrence and progression of certain diseases. In neurological disorders such as Rett syndrome [49], abnormal alterations in 5hmC levels have been observed, correlating with disease mechanisms and progression. Collectively, these research findings underscore the pivotal role of 5hmC in the genome and epigenetic modifications.

## 6. Multiple Functions of 5hmC

### 6.1. The Role of 5hmC in Transcriptional Regulation

**Figure 3:**
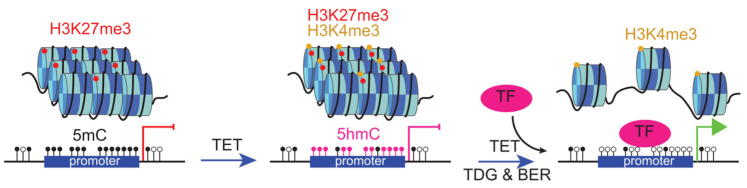
5hmC plays a crucial role in transcription. Bivalent promoters contain both H3K27me3 and H3K4me3 modifications. H3K27me3 is associated with gene silencing and can block the binding of transcription factors (TFs). However, after undergoing the TET enzyme and TDG–BER pathway, TFs can bind to the promoter region, initiating the process of gene transcription.

Firstly, 5hmC exerts a regulatory role in gene promoters. The enrichment of 5hmC in promoters demonstrates its ability to facilitate demethylation and activation processes, thereby exerting a significant impact on transcriptional activity and the transcriptional status of genes [50]. Employing high-throughput sequencing of 5hmC-containing DNA derived from mouse embryonic stem cells (ESCs), one study revealed a pronounced enrichment of 5hmC at bivalent promoters distinguished by the concurrent presence of dual histone modifications, namely H3K27me3 and H3K4me3 [51]. Bivalent promoters are characterized by the coexistence of H3K4me3, an active gene marker, and H3K27me3, a repressive gene marker. Additionally, these promoters often exhibit a poised and balanced state, primed for transcriptional activity [52] (Figure 3). This bivalent characteristic allows these genes to either be rapidly activated or permanently silenced in response to specific signals. During transcription, transcription factors (TFs) bind to bivalent promoters, leading to the removal of H3K27me3 marks and the retention of H3K4me3 marks. Meanwhile, the TDG–BER pathway facilitates the removal of methylation-associated repressive marks, further promoting gene activation. Together, these mechanisms enable the precise and timely expression of specific developmental genes in response to the appropriate cellular cues [53].

Secondly, 5hmC also plays a crucial role in regulating transcriptional regulatory regions. Through genome-wide and single-base resolution mapping of 5hmC and 5mC in the human brain, our study revealed a robust enrichment of 5hmC in gene regions and distal regulatory elements. Particularly, we observed a higher enrichment of 5hmC in potential enhancers compared to active enhancers, underscoring its involvement in enhancer-mediated regulation [54]. Consistent with its localization in enhancers, 5hmC exhibited significant enrichment in histone modifications associated with enhancers, such as H3K4me1 and H3K27ac [55]. Furthermore, we observed enrichment of 5hmC in other protein–DNA interaction sites, including binding sites for OCT4 and NANOG, highlighting its potential role in modulating transcription factor activity [56]. Notably, 5hmC demonstrated distinctive characteristics in transcription factor binding sites, with low levels of 5mC and high levels of 5hmC observed in key transcription factors such as CTCF and AR, particularly in embryonic stem cells [57].

Thirdly, 5hmC exhibits interactions with specific binding proteins, primarily transcriptional regulatory factors. Notably, there is minimal overlap between the proteins recognizing 5mC and 5hmC, with proteins binding to oxidized forms of cytosine showing enrichment in repair proteins and transcriptional regulatory factors. Through a comparative analysis of the enrichment of various DNA-binding proteins with distinct affinities for CpG sites, only a select few proteins demonstrated a preference for 5hmC, including RPL26, PRP8, and MHS6 [58]. MeCP2, for instance, exhibits the ability to bind to both 5mC and 5hmC, thereby modulating chromatin structure and facilitating gene expression within cells [49]. Conversely, MBD1 specifically binds to 5mC but not 5hmC [59]. Intriguingly, the interplay between MeCP2 and 5hmC may contribute to a cell-specific epigenetic regulatory mechanism involved in the modulation of chromatin structure and gene expression.

Many proteins specifically recognize and bind to 5hmC. The TET enzyme family is responsible for converting 5mC into 5hmC, playing a crucial role in subsequent demethylation processes. MeCP2, which is highly expressed in the nervous system, can recognize and bind to 5hmC, thereby regulating neuronal development and function. YTHDC1, an RNA-binding protein, influences RNA fate and functionality within the cell by recognizing and binding to 5hmC [60]. TGF-β inducible early gene-1 (TIEG1), through its recognition of 5hmC, can either promote or inhibit the expression of specific genes, thus affecting cellular biological functions and fates [61]. Through the recognition and binding of 5hmC, these proteins contribute to the regulation of gene expression, cellular differentiation, and epigenetic mechanisms, further highlighting the relevance of 5hmC as an important epigenetic mark.

Lastly, 5hmC is associated with chromatin structure alterations. It is highly enriched in euchromatin, especially in active gene regions, in Purkinje neurons [62], suggesting a potential role of 5hmC in promoting gene transcription within euchromatin. On the other hand, heterochromatin is enriched in 5mC and tends to be associated with gene-silencing regions [63]. High levels of 5hmC are observed in diffuse nuclear speckles, consistent with the enrichment of 5hmC in euchromatin [55]. The distribution of these two forms of DNA methylation is also associated with corresponding histone marks: 5mC, along with repressive H3K9me3 and H3K27me3, is predominantly located in heterochromatin regions, while 5hmC, along with H3K4me2, is mainly found in euchromatin regions [64]. Overall, 5hmC plays an important role in transcriptional regulation, with significant implications for cellular function and developmental processes.

### 6.2. The Role of 5hmC in Cell Fate Determination

Epigenetic variations have long been recognized as influential factors shaping the functional diversity of embryonic stem cells (ESCs) and induced pluripotent stem cells (iPSCs), thereby modulating the response of signaling pathways involved in developmental trajectories and promoting biases in cellular differentiation [65]. Key transcription factors, such as NANOG, are believed to govern the molecular control of pluripotency, and recent studies have demonstrated the co-occupancy of genomic loci associated with pluripotency maintenance and differentiation by TET1 and NANOG in embryonic stem cells [66]. Additionally, a synergistic interplay between TET1, TET2, and NANOG has been revealed, leading to enhanced reprogramming efficiency [66]. Oct4, a pivotal transcription factor regulating self-renewal and differentiation processes in embryonic stem cells, exhibits the ability to sustain stem cells in an undifferentiated state. Intriguingly, investigations have unveiled that TET1 can act as a substitute for Oct4, collaborating with Sox2, Klf4, and c-Myc to initiate the reprogramming of somatic cells [67]. Moreover, NANOG can recruit TET1 to facilitate the expression of critical target genes during reprogramming [66].

During the initiation of pluripotent reprogramming in embryonic germ cells (EGCs), rapid re-expression of Oct4 coincides with TET1-mediated oxidation of 5mC at multiple imprint control regions (ICRs). Furthermore, the presence of TET2 is crucial for the effective reprogramming of EGCs, indicating distinct roles for TET1 and TET2 in pluripotent reprogramming [68]. In the early stages of cellular reprogramming, TET2 is recruited to responsive gene loci as early as the fourth day after c-Myc induction, playing a pivotal role in the initial oxidation of 5mC to 5hmC [69]. Several studies have suggested that the absence of TET1 results in slightly smaller mouse size, while a partial decrease in 5hmC levels does not impact the pluripotency of ESCs [70]. Depletion of TET2 leads to delayed differentiation of hematopoietic stem cells (HSCs) and biases development toward the monocyte/macrophage lineage [71]. Moreover, TET1 regulates the lineage differentiation potential of ESCs, as TET1-deficient ESCs exhibit increased endoderm, decreased neuroectoderm, and ectopic appearance of trophoblast giant cells [72]. These findings provide compelling evidence that 5hmC is an epigenetic modification associated with pluripotency and its presence is linked to the reprogramming of mouse fibroblasts into induced pluripotent stem cells (iPSCs) [73]. Overall, 5hmC, as a significant epigenetic modification, holds profound implications for cellular function, disease occurrence, and development, and its research value and potential are yet to be fully explored and unveiled.

### 6.3. The Role of 5hmC in Embryonic Development

There are three members in the TET family of mice, TET1, TET2 and TET3. In mouse embryonic stem cells (ESCs), TET1 exhibits a high expression level, approximately five times higher than TET2, whereas TET3 expression is considerably low [74]. However, throughout the differentiation process of ESCs, the expression levels of TET1 and TET2 undergo a significant decrease, while TET3 expression shows an increase [75]. These findings strongly suggest a tight correlation between TET1 and TET2-mediated DNA demethylation and the maintenance of embryonic stem cells.

The functional roles of TET enzymes can be investigated through gene silencing or knockout strategies. In mouse ESCs, the knockout of the TET1 gene results in reduced levels of 5hmC and minor alterations in global gene expression. Additionally, it leads to a biased differentiation towards the trophectoderm lineage in vitro [76]. Loss of TET1 function also diminishes the self-renewal capacity of adult neural stem cells [77]. Deletion of TET2 causes a significant decrease in 5hmC levels on enhancers, accompanied by enhancer hypermethylation, reduced enhancer activity, and delayed gene induction during early-stage cellular differentiation [78]. These findings indicate that while the individual knockout or silencing of TET1 and TET2 leads to reduced 5hmC levels, it does not severely impair pluripotency and development in mice. In the case of TET3, its deficiency impedes the demethylation process of paternal Oct4 and Nanog genes, resulting in a delay in the subsequent activation of the paternal Oct4 transgene in early embryos. This delay occurs because TET3-mediated DNA hydroxymethylation is involved in the epigenetic reprogramming of paternal DNA following natural fertilization [79].

In the case of TET1 and TET2 double knockout (TET1/2 DKO) mice, embryonic stem cells (ESCs) maintain their pluripotency but exhibit a deficiency in 5hmC, leading to defects in chimeric embryo development. While some double-mutant embryos present mid-gestation abnormalities and experience perinatal death, we also observe the generation of viable and visibly normal TET1/2 DKO mice. The DKO mice display reduced levels of 5hmC, elevated levels of 5mC, and abnormal methylation patterns at various imprinting sites. Interestingly, the data also suggest a significant involvement of TET3 in 5mC hydroxymethylation during development [80]. Moreover, embryos lacking both TET1 and TET3 (TET1/3 DKO) exhibit a substantial loss of 5hmC at the eight-cell stage. In addition, these embryos show aberrant gene expression in individual cells of eight-cell embryos and individual blastocysts on day 3.5, which aligns with increased variability of 5mC/5hmC in gene bodies and repetitive elements compared to controls [81].

To assess the developmental potential, we generated TET1/2/3 triple-knockout (TKO) mouse embryonic stem cells (ESCs), uncovering that the simultaneous absence of all three TET enzymes results in the depletion of 5hmC and impairs ESC differentiation. This impairment is evident in the form of poorly differentiated TKO embryoid bodies (EBs) and teratomas. Through whole-genome expression and methylation analysis, we observe promoter hypermethylation and dysregulation of genes associated with embryonic development and differentiation [82]. Notably, TET-deficient mouse embryonic fibroblasts (MEFs) fail to undergo reprogramming through the mesenchymal-to-epithelial transition (MET) step, and the reprogramming of MEFs lacking TDG is also affected [83]. Additionally, TET TKO ESCs demonstrate an increased occurrence of telomeric sister chromatid exchange and elongated telomeres [84]. Collectively, these studies underscore the critical role of TET-mediated DNA demethylation in preserving the integrity of ESCs during differentiation and embryonic development.

### 6.4. The Role of 5hmC in Neurodevelopment

The role of 5hmC and the TET family in the differentiation of ESCs, particularly in the process of neuronal differentiation, has garnered considerable attention among researchers. Numerous research reports have provided evidence for the involvement of TET proteins in the nervous system. Studies have demonstrated that alterations in 5hmC levels can profoundly impact gene expression patterns, thereby influencing the fate determination and cellular function of neural stem cells. Moreover, 5hmC has been observed to be intricately associated with the development and maturation of the nervous system, exerting a significant regulatory role in neuronal formation, synapse establishment, and functional modulation.

Similarly, 5hmC plays a pivotal role in neuronal development and differentiation. TET1 emerges as a key player in the proliferation of neural precursor cells (NPCs) in the adult mouse brain, as evidenced by the hippocampal neural damage and impaired learning and memory abilities observed in TET1 knockout mice [85]. In adult NPCs lacking TET1, a specific group of genes involved in precursor cell proliferation undergoes hypermethylation and downregulation, suggesting a positive contribution of TET1 to the epigenetic regulation of adult brain NPC proliferation [86]. Notably, overexpression of TET1 leads to the upregulation of multiple genes associated with neural memory, while overexpression of a catalytically inactive mutant variant (TET1m) results in impaired contextual fear memory, indicating that TET1’s enzymatic activity is crucial for normal memory function [87]. Furthermore, depletion of TET2 results in enhanced proliferation of adult neural stem cells and diminished differentiation both in vitro and in vivo [88]. While TET3 gene knockout ESCs exhibit normal self-renewal and maintenance properties, they are compromised in terms of neuronal differentiation. Although NPCs can be efficiently induced from TET3 gene knockout ESCs, they undergo rapid apoptosis, resulting in a significant reduction in the terminal differentiation of neurons [89].

The dynamic distribution of 5hmC in neurons strongly suggests its functional significance. Investigation of the patterns of 5mC and 5hmC during mouse embryonic brain neurogenesis revealed an increase in 5hmC levels during neuronal differentiation, with a notable association between 5hmC and genes involved in activated neural functions. Remarkably, the formation of 5hmC appears to cooperate with the loss of H3K27me3 to facilitate brain development [90]. Through genome-wide profiling of 5hmC in the hippocampus and cerebellum of mice at three distinct developmental stages, the stability and dynamic regulation of postnatal neurodevelopment into adulthood were assessed. The findings demonstrated that 5hmC in neural cells undergoes developmental programming, and the epigenomic profiling of 5hmC regulatory regions revealed sites that undergo stable and dynamic modifications during neural development and aging processes [48]. The dynamic changes exhibited by 5hmC at various stages of nervous system development strongly suggest its regulatory role in neuronal development. Consequently, these studies collectively provide compelling evidence for the critical involvement of 5hmC in neuronal fate determination, neural stem cell self-renewal, and the overall processes of neural development and maturation.

### 6.5. The Role of 5hmC in Diseases Development

Abnormal and dysregulated DNA methylation is intricately linked to cancer, with the malfunctioning of TET enzymes causing a widespread reduction in DNA methylation levels. This disruption significantly influences the expression of pivotal genes and cellular phenotypic traits. Consequently, these alterations can profoundly impact tumor stem cell properties, proliferation potential, and cellular differentiation, potentially leading to chromosomal instability and aberrant remodeling of the genomic DNA. Ultimately, these molecular perturbations amplify the risk of cellular carcinogenesis.

Aberrant expression of TET enzymes and 5hmC has been widely observed in various tumors, cancers, and hematological disorders. In Haferlach leukemia samples, overexpression of TET1 and TET2 was identified, whereas TET3 downregulation was noted in Andersson leukemia samples [91]. Squamous cell lung cancer exhibited a significant decrease in 5hmC levels compared to normal lung tissue, while brain tumors displayed a more pronounced reduction, with levels more than 30-fold lower than normal brain tissue [92]. In-depth analysis of melanoma’s epigenomic landscape uncovered a comprehensive loss of 5hmC, and the reintroduction of active TET2 or isocitrate dehydrogenase 2 (IDH2) in melanoma cells restored the 5hmC landscape and suppressed melanoma growth [93]. Notably, TET enzymes play a crucial role in balancing miR-200 and miR-22, thereby influencing the metastatic properties and stem cell characteristics of breast cancer cells [94]. Somatic mutations in TET2 are frequently observed in various myeloid malignancies, including myelodysplastic syndromes (MDS), myeloproliferative neoplasms (MPN), and MDS/MPN overlap syndromes, including chronic myelomonocytic leukemia (CMML), acute myeloid leukemia (AML), and secondary AML (sAML) [95]. These findings underscore the critical importance of TET2 in normal hematopoiesis and suggest that disruption of TET2 enzyme activity favors the development of myeloid malignancies. Moreover, TET2 (+/−) mice exhibited increased self-renewal of stem cells and extramedullary hematopoiesis, providing evidence that TET2 haploinsufficiency contributes to hematopoietic transformation in vivo [96].

Aberrant levels of 5hmC have been implicated in neurological disorders and diseases. In an Alzheimer’s disease (AD) mouse model (5xFAD), loss of TET1 resulted in a notable increase in amyloid plaque burden and displayed a non-significant trend towards exacerbating AD-related stress responses [97]. Genome-wide profiling of Parkinson’s disease (PD) patients’ substantia nigra unveiled substantial alterations in 5hmC levels, including the presence of PD-specific differentially hydroxymethylated regions (DhMRs) [98]. Remarkable changes in 5hmC have also been identified in conditions such as schizophrenia [99], autism spectrum disorders (ASDs) [100], and broader psychiatric disease genes. These findings shed light on the potential involvement of 5hmC in the pathogenesis of these disorders, contributing to a deeper understanding of their underlying molecular mechanisms.

Moreover, TET enzymes have been implicated in various other diseases. For instance, the knockout of TET2/TET3 induces noticeable changes in hair shape and length, ultimately leading to hair loss [101]. Studies have demonstrated that the loss of MECP2 results in decreased expression of TET enzymes and a subsequent loss of 5hmC, potentially contributing to neurological dysfunction and developmental stagnation observed in Rett syndrome patients [49]. Additionally, abnormal expression of 5hmC has been associated with cardiovascular diseases, diabetes [102], autoimmune diseases, and other disorders. These findings suggest that 5hmC may play pivotal roles in the pathogenesis of these conditions, facilitating a deeper comprehension of the underlying molecular mechanisms and pathophysiological processes involved in disease onset and progression. Such knowledge holds the potential to unveil novel therapeutic targets and mechanisms, paving the way for advancements in fundamental scientific research.

## 7. 5hmC Is a Promising Epigenetic Marker for Disease Diagnosis, Treatment, and Prognosis

Due to its association with various diseases and their progression, 5hmC emerges as a promising epigenetic marker for disease diagnosis, treatment, and prognosis. The levels of 5hmC exhibit distinct patterns of alterations across different diseases, thereby serving as a potential biomarker for disease diagnosis and classification. Immunohistochemical analysis demonstrates a significant reduction in 5hmC levels in numerous human cancers, establishing an inverse relationship between 5hmC levels and cell proliferation, thereby suggesting its utility as a valuable biomarker for cancer diagnosis [103]. Moreover, the dynamic and abnormal changes in 5hmC levels hold the potential to serve as indicative markers for diseases associated with abnormal cell proliferation, including liver cancer, pancreatic cancer [104], neurological disorders such as Alzheimer’s disease and Parkinson’s disease [105], as well as autoimmune diseases like rheumatoid arthritis and systemic lupus erythematosus [106].

5hmC has been elucidated as a cellular state indicator during organ maturation and drug-induced responses. Targeting TET1 and its associated pathways emerges as a promising therapeutic strategy, offering potential in mitigating the adverse effects of environmental exposure and preventing or treating exposure-related diseases [107]. Likewise, the utilization of 5hmC analysis as an indicator of cellular state during organ maturation and drug-induced responses has been proposed, providing novel epigenetic features for non-genotoxic carcinogen exposure [108]. Notably, changes in 5hmC levels have been identified as biomarkers for the diagnosis, treatment monitoring, and recurrence surveillance of liver cancer. Researchers have demonstrated the utility of extracellular 5hmC sequencing combined with hepatocellular carcinoma (HCC) scoring in detecting HCC, monitoring treatment outcomes, and assessing recurrence status [109].

Multiple studies have consistently demonstrated a correlation between reduced 5hmC levels and increased tumor invasiveness, indicating the potential of 5hmC as a predictive marker for metastasis, recurrence, and prognosis. In the context of non-small cell lung cancer (NSCLC), 5hmC levels have been identified as an independent prognostic factor for overall survival, with downregulation of 5hmC serving as a promising biomarker for assessing NSCLC prognosis [110]. Furthermore, deficiencies in Fe(II), ascorbic acid, or oxygen have been shown to attenuate the activity of TET enzymes. As a therapeutic approach, supplementation of these substances has been explored. Genome-wide analysis of 5hmC in tumor tissues and vitamin C-treated bladder cancer cells has revealed that vitamin C treatment elevates 5hmC levels, induces transcriptomic alterations, and inhibits the malignant phenotypes associated with bladder cancer cell lines in vitro, as well as xenografts in vivo [111].

In addition to its role as a biomarker for disease diagnosis and classification, 5hmC exhibits potential as a novel epigenetic therapeutic strategy. The alterations in 5hmC levels, whether loss or gain, associated with disease prognosis, may also correlate with patient survival and disease progression. Assessing the relationship between 5hmC levels and disease prognosis can be achieved through survival analysis of patient cohorts and long-term follow-up observations, providing valuable insights for patient treatment and management. However, it is important to note that 5hmC itself may not serve as a primary diagnostic biomarker for different diseases; rather, it functions more as an auxiliary tool. Effective diagnosis often requires more specific biomarkers to achieve conclusive results. Overall, 5hmC, as a significant epigenetic marker, holds promise in disease diagnosis, treatment, and prognosis. However, comprehensive and systematic exploration is necessary across different disease types and individuals to fully elucidate the precise mechanisms and clinical applications of 5hmC in disease onset and progression.

## 8. Conclusions

**Figure 4:**
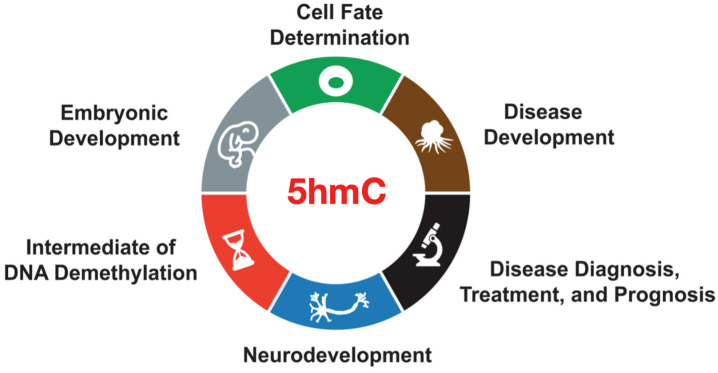
The functions of 5hmC. 5hmC is an intermediate product of DNA demethylation, and it also plays roles in embryonic development, cell fate determination, neurodevelopment, disease development, disease diagnosis, treatment, and prognosis.

Currently, there is a substantial body of researchers focused on 5hmC as a crucial epigenetic modification implicated in various biological functions. These studies have provided compelling evidence that 5hmC plays a pivotal regulatory role, not only in shaping gene expression patterns and determining cell fate during cell differentiation [48], but also in profoundly influencing the trajectory and tempo of embryonic development during gene reprogramming [68]. Additionally, 5hmC is actively involved in the process of stem cell differentiation into neurons, exerting an impact on neuronal development and maturation [85]. Moreover, investigations have established connections between 5hmC and specific neurodegenerative diseases as well as cancer, offering novel insights and potential targets for the diagnosis and treatment of these conditions [91] (Figure 4).

Despite significant advancements in comprehending the mechanisms and functions of active DNA demethylation and 5hmC, numerous unanswered inquiries persist. Firstly, the current methods employed for detecting 5hmC exhibit limitations and necessitate more precise and sensitive approaches to minimize both false positives and false negatives. Secondly, dynamic alterations in 5hmC have been observed across diverse cell types, developmental stages, and environmental conditions, yet our understanding of the temporal scale, specific patterns, and regulatory factors governing these dynamic changes remains limited. Thirdly, while the connection between 5hmC and certain diseases (such as neurodegenerative disorders and cancer) has been established, further investigation is required to elucidate the precise mechanisms and regulatory networks underlying these associations. Lastly, in addition to TET enzymes, the mechanisms and regulatory networks of other factors that potentially influence 5hmC levels, including DNA methyltransferases, DNA repair enzymes, and DNA-binding proteins, necessitate more extensive research.

In the future, comprehensive efforts can be directed towards gaining a deeper understanding of the mechanisms underlying the biological functions and disease associations of 5hmC, thereby offering new insights and advancements in related research and clinical translation. Firstly, there is a scope for developing novel bioinformatics tools and algorithms to integrate and analyze large-scale 5hmC data, facilitating the exploration of associations between 5hmC and genomic functions, as well as regulatory networks. Secondly, the investigation of distribution patterns and functional significance of 5hmC in the tumor genome can be accomplished by integrating extensive 5hmC sequencing data with tumor genomic data. Moreover, studying the role of 5hmC in the regulation of tumor suppressor genes and oncogenes can shed light on its dual function in tumor suppression and promotion. Additionally, the development of new therapeutics for tumor therapy, based on the functional modulation of enzymes associated with 5hmC, holds promise for exploring innovative treatment strategies and drug targets. Collectively, these collaborative endeavors will contribute to a more profound comprehension of the pivotal role of 5hmC in gene expression regulation, cell fate determination, and other vital biological processes. Furthermore, they will enhance our understanding of the complex regulatory networks of 5hmC implicated in the occurrence and progression of diseases, providing a robust theoretical foundation and clinical applicability for future research in relevant fields.

## Data Availability

Not applicable.
